# Risk factors for flow-related aneurysm rupture associated with posterior circulation arteriovenous malformation: a multicenter retrospective study

**DOI:** 10.3389/fneur.2025.1685261

**Published:** 2025-11-24

**Authors:** Wang Yifei, Li Guangxi, Li Chenyuan, Chen Shu, Chekera Ropafadzo Leocardia, Chang Hanxiao, Luo Han, Li Wenyan, Li Zheng, Lin Chao, Xu Xiupeng, Chen Zhi, Li Wen, Lu Hua

**Affiliations:** 1 Department of Neurosurgery, Jiangsu Province Hospital and the First Affiliated Hospital with Nanjing Medical University, Nanjing, Jiangsu, China; 2 Department of Neurosurgery, The First Affiliated Hospital of Soochow University, Suzhou, Jiangsu, China; 3 Department of Neurosurgery, Southwest Hospital, Third Military Medical University (Army Medical University), Chongqing, China

**Keywords:** intracranial aneurysm, arteriovenous malformation, posterior circulation, subarachnoid hemorrhage, flow-related aneurysm

## Abstract

**Background:**

Flow-related aneurysms (FAs) are more common and have an increased risk of rupture in posterior circulation arteriovenous malformations (pAVMs). However, the risk factors for FA rupture in pAVMs are understudied and warrant further investigation.

**Objective:**

This study aimed to investigate the risk factors for FA rupture associated with pAVMs using a multi-centric database from 1 January 2020 to 31 December 2024.

**Methods:**

Patients diagnosed with AVMs were selected from databases. Patients with ruptured FAs and those with unruptured FAs were compared. Independent Student’s *t*-test, Mann–Whitney *U*-test, Fisher’s exact test, and multivariable binary logistic regression were used for analysis.

**Results:**

Among 284 patients, 37 patients with 22 ruptured FAs and 28 with unruptured FAs met the inclusion criteria. The results indicated that ruptured FAs were associated with smaller pAVMs (2.41 ± 1.02 cm vs. 3.93 ± 2.68 cm, *p* = 0.001) and Spetzler–Martin grades I to II (*p* = 0.013). Patients with ruptured FAs had greater aneurysm size [median size, 4.55 mm (IQR, 3.20–7.55 mm) vs. 3.00 mm (IQR, 2.40–5.00 mm); *p* = 0.009], larger size ratio [median, 2.72 (IQR, 1.95–4.12) vs. 2.23 (1.10–2.87); *p* = 0.021], and larger relative size ratio [median, 0.24 (IQR, 0.137–0.358) vs. 0.096 (IQR, 0.063–0.129); *p* < 0.001] than those with unruptured FAs. The multivariable binary logistic regression analysis indicated that only the AVM size [OR, 0.92 (95% CI, 0.84–0.99); *p* = 0.039] and the size ratio [OR, 1.66 (95% CI, 1.07–2.59); *p* = 0.024] remained associated with the risk of rupture.

**Conclusion:**

The size of pAVMs and size ratio of FAs to average diameter of the parent vessel are independent risk factors for FA rupture associated with pAVMs.

## Introduction

Intracranial aneurysms associated with brain arteriovenous malformations (AVMs) can be classified into three types: unrelated aneurysms (type I), flow-related aneurysms (type II), and intranidal aneurysms (type III) (1). Aneurysm rupture constitutes the primary cause of hemorrhage in AVMs with associated aneurysms, accounting for approximately 49.2% of all cases (2). Flow-related aneurysms (FAs), with an overall prevalence of 11.2—25%, significantly increase the hemorrhagic risk, particularly when located in the posterior circulation (1–5).

Multiple risk factors for intracranial aneurysm rupture have been established in previous studies, including hypertension, smoking, and morphological features such as irregular shape, aspect ratio ≥1.3, and elevated size ratio (6–10). Older age, smaller AVM size, and cerebellar location have been identified as risk factors for FA rupture in a retrospective study involving 69 FA cases (11). In contrast, another study of 101 FAs found that hypertension and high-grade AVMs (Spetzler–Martin grades III–V) were associated with bleeding risk in univariate analysis, with only hypertension remaining significant in multivariable analysis (12).

Despite this existing evidence, risk factors specifically for FA rupture remain poorly defined; however, understanding these factors are critical for informed clinical decision-making. In this study, we aim to identify the predictors of rupture in posterior circulation AVMs (pAVMs) with associated FAs, analyzing demographic, angioarchitectural, and morphological characteristics.

## Methods

### Patient population

This is a retrospective, observational study approved by the institutional review committee (2024-SR-777) and registered on ClinicalTrials.gov (NCT07050381). The study included patients diagnosed with pAVMs and associated FAs by digital subtraction angiography (DSA) at Jiangsu Province Hospital, The First Affiliated Hospital of Soochow University, and Southwest Hospital from 1 January 2020 to 31 December 2024. Patients were excluded from the study if they lacked clinical or DSA data or had a dissecting aneurysm.

### Variable definition

**Figure 1:**
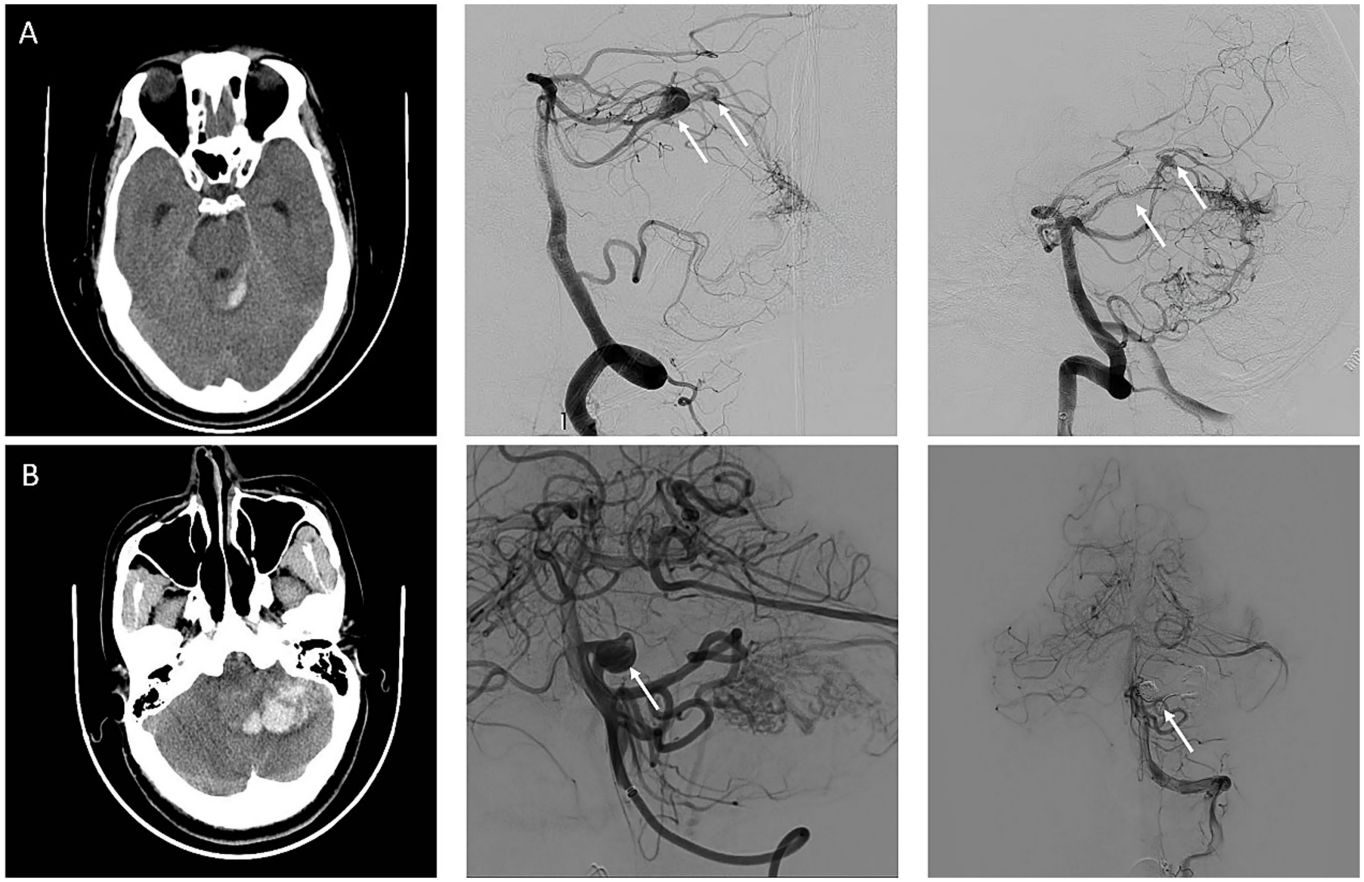
Representative cases of ruptured flow-related aneurysm and unruptured flow-related aneurysm. **(A)** An adult patient with a sudden headache was diagnosed with subarachnoid hemorrhage on a computed tomography (CT). Arteriovenous malformation fed by the left superior cerebellar artery and two flow-related aneurysms (white arrow) were detected on digital subtraction angiography. The larger FAs with a maximum diameter of 10 mm were the reason for hemorrhage and was treated with coils for the first time. **(B)** An adult patient diagnosed with intracerebral hemorrhage due to immediate headache and imbalance by CT. AVMs fed by the left anterior inferior cerebellar artery and posterior inferior cerebellar artery and FAs (white arrow) located in the anterior inferior cerebellar artery were detected in DSA. FAs and AVMs were treated with coils and onyx at the same time.

Demographic and clinical information included age, sex, hypertension, and diabetes history. The initial diagnosis of FA rupture was conducted when a non-contrast CT scan at admission demonstrated isolated subarachnoid hemorrhage (SAH) without concurrent intracerebral hemorrhage (ICH) (Figure 1). For cases with a mixed pattern of hemorrhage on CT, the diagnosis was based on a comprehensive assessment based on two key aspects: the anatomical correlation between the hemorrhage location and the lesion and the characteristics of the AVMs and FAs observed on intraoperative 3D-DSA reconstruction. The radiographic variables of AVMs included their size, location, Spetzler–Martin grade, and the presence of deep venous drainage. FA size, location, aspect ratio (AR, aneurysm depth to aneurysm neck (13)), size ratio (SR, aneurysm maximum diameter to average parent vessel diameter (10)), and relative size ratio (RSR, aneurysm maximum diameter to AVM maximum diameter) were collected to assess the characteristics of FAs. Angioarchitectural characteristics were recorded using 3D-DSA. Additionally, the therapeutic approach and treatment sequence were recorded. Modified Rankin scale (mRS) was used to evaluate the functional status at presentation and follow-up.

### Statistical analysis

Continuous variables are expressed as mean ± standard deviation (SD) or median (interquartile range, IQR) based on distribution normality. Categorical variables are shown in numbers (percentages). Independent Student’s *t*-test and Mann–Whitney *U*-test were used for analyzing normally and non-normally distributed variables, respectively. Fisher’s exact test was used to analyze categorical variables. Multicollinearity was assessed via variance inflation factors (VIFs) (14). Variables with VIF ≥10 were excluded from the multivariable model. Multivariable binary logistic regression analysis was performed to assess the correlation of multiple factors influencing FA rupture. A two-sided *p*-value of <0.05 is considered statistically significant. All data were analyzed using SPSS (version 26.0; IBM).

## Results

### Baseline demographic and clinical information

**Figure 2:**
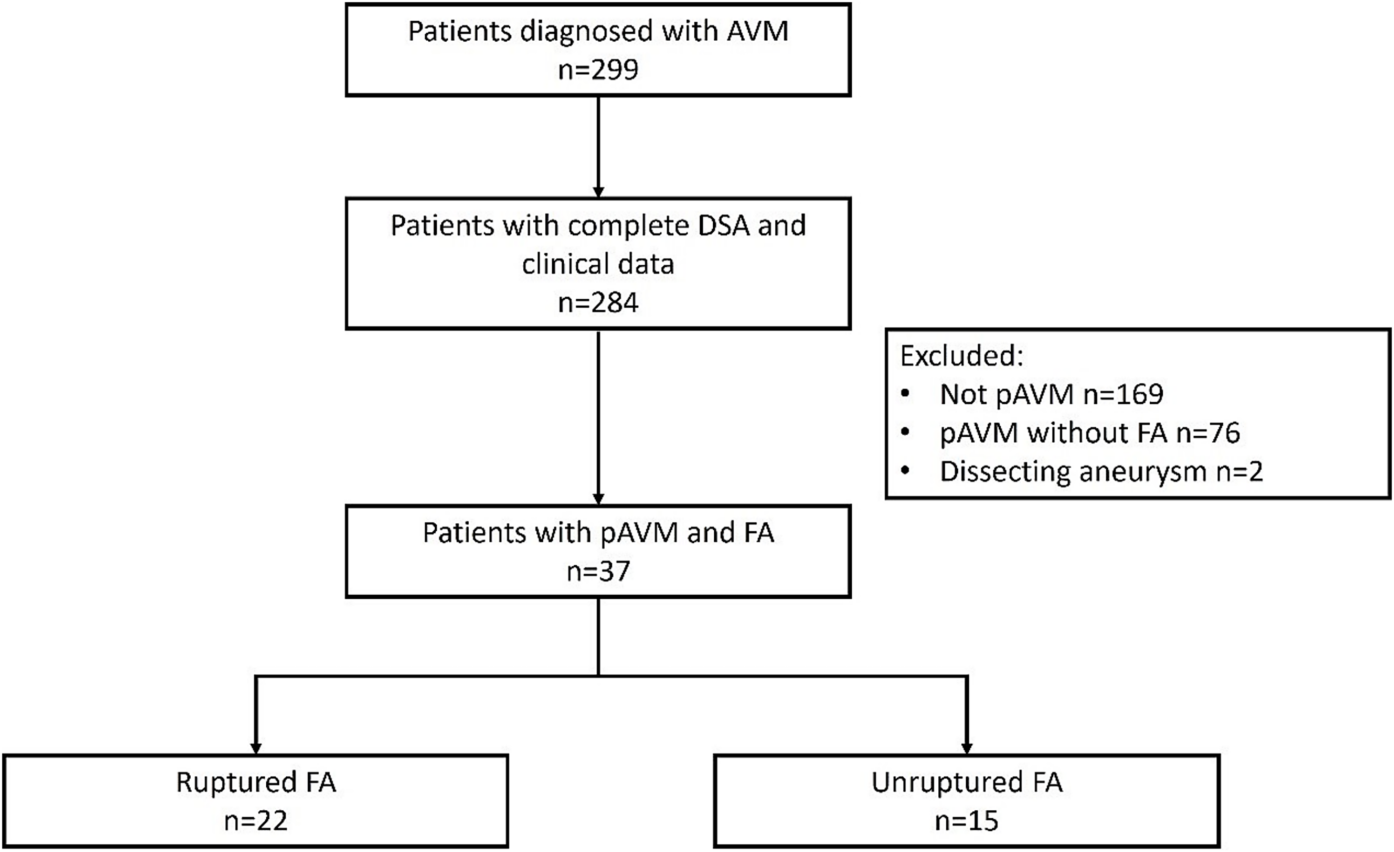
Flow chart of patient selection.

A total of 299 patients diagnosed with AVMs were selected from the database. Of these, 284 patients had complete DSA and clinical data, while 115 (40.5%) patients were diagnosed with pAVMs. Moreover, 76 patients did not have FAs, and 2 had dissecting aneurysms. Ultimately, 37 (32.7%) patients with associated FAs were included for analysis, and 22 (59.4%) patients with hemorrhagic presentations experienced these due to ruptured FAs (Figure 2). Baseline demographics are shown in Table 1. FAs are more likely seen in men (78.4%), and the mean age was 47.35 ± 15.62 years. No significant differences were found in terms of age (49.18 ± 14.04 vs. 44.67 ± 17.85, *p* = 0.396), sex (*p* = 0.690), hypertension (36.4% vs. 33.3%, *p* > 0.999), or diabetes (9.1% vs. 13.3%, *p* > 0.999) between patients with pAVMs who had ruptured versus unruptured FAs.

**Table 1:** Demographic and clinical information of patients with ruptured and unruptured flow-related aneurysms.

Parameters	Total (*n* = 37)	Ruptured FAs (*n* = 22)	Unruptured FAs (*n* = 15)	*p*-value
Age, year, mean (SD)	47.35 (15.62)	49.18 (14.04)	44.67 (17.85)	0.396
Sex, male patients, *n* (%)	29 (78.4)	18 (81.8)	11 (73.3)	0.690
Hypertension, *n* (%)	13 (35.1)	8 (36.4)	5 (33.3)	>0.999
Diabetes, *n* (%)	4 (10.8)	2 (9.1)	2 (13.3)	>0.999

### Characteristics of pAVMs and FAs

Characteristics of pAVMs and FAs are shown in Table 2. The average pAVM nidus size in maximum diameter was 2.41 ± 1.02 cm and 3.93 ± 2.68 cm for ruptured FAs and unruptured FAs, respectively (*p* = 0.001). The AVMs with ruptured FAs tend to have a lower Spetzler–Martin grade (grade 1 and 2) with a significant difference (*p* = 0.013). The eloquence location (18.2% vs. 40%, *p* = 0.258), deep venous drainage (45.5% vs. 46.7%, *p* > 0.999), and AVM location (*p* = 0.334) were found to be similar between pAVMs with ruptured FAs and unruptured FAs.

**Table 2:** Radiographic variables of posterior circulation arteriovenous malformations and flow-related aneurysms.

Radiographic variables of pAVMs				
Parameters	Total (*n* = 37)	Ruptured FAs (*n* = 22)	Unruptured FAs (*n* = 15)	*p*-value
AVM size, cm, mean (SD)	3.02 (2.23)	2.41 (1.02)	3.93 (2.68)	0.001^a^
Spetzler–Martin grading, *n* (%)				0.013^a^
Grade 1	11 (29.7)	8 (36.4)	3 (20)	
Grade 2	12 (32.4)	10 (45.5)	2 (13.3)	
Grade 3	11 (29.7)	4 (18.2)	7 (46.7)	
Grade 4	3 (8.1)	0 (0)	3 (20)	
Grade 5	0 (0)	0 (0)	0 (0)	
Eloquence, *n* (%)	10 (27.0)	4 (18.2)	6 (40.0)	0.258
Deep venous drainage, *n* (%)	17 (45.9)	10 (45.5)	7 (46.7)	>0.99
Location				0.334
Occipital	9 (24.3)	3 (13.6)	6 (40)	
Cerebellar hemisphere	21 (56.8)	13 (59.1)	8 (53.3)	
Cerebellar vermis	2 (5.4)	2 (9.1)	0 (0)	
Brain stem	2 (5.4)	2 (9.1)	0 (0)	
Other	3 (8.1)	2 (9.1)	1 (6.7)	

Radiographic variables of FAs				
Parameters	Total (*n* = 50)	Ruptured FAs (*n* = 22)	Unruptured FAs (*n* = 28)	*p*-value
Aneurysm size, mm, median (IQR)	3.75 (2.60–6.42)	4.55 (3.20–7.55)	3.00 (2.40–5.00)	0.009^a^
Location, *n* (%)				0.652
Posterior cerebral artery	13 (26.0)	4 (18.2)	9 (32.1)	
Superior cerebellar artery	16 (32.0)	9 (40.9)	7 (25.0)	
Anterior inferior cerebellar artery	9 (18.0)	4 (18.2)	5 (17.9)	
Posterior inferior cerebellar artery	8 (16.0)	4 (18.2)	4 (14.3)	
Basilar artery	4 (8.0)	1 (4.5)	3 (10.7)	
AR, median (IQR)	1.16 (1.00–1.78)	1.16 (1.03–1.85)	1.17 (1.00–1.48)	0.506
SR, median (IQR)	2.37 (1.24–3.78)	2.72 (1.95–4.12)	2.23 (1.10–2.87)	0.021^a^
RSR, median (IQR)	0.129 (0.076–0.268)	0.24 (0.137–0.358)	0.096 (0.063–0.129)	<0.001^a^

AR, aspect ratio; SR, size ratio; RSR, relative size ratio.

a Significant variables (*p* < 0.05).

A total of 50 FAs were detected in 37 patients. Larger aneurysm size was correlated with FA rupture [median size, 4.55 mm (IQR, 3.20–7.55 mm) vs. 3.00 mm (IQR, 2.40–5.00 mm); *p* = 0.009]. The ruptured group had a bigger SR than the unruptured group [median, 2.72 (IQR, 1.95–4.12) vs. 2.23 (1.10–2.87); *p* = 0.021]. A larger RSR was also associated with FA rupture [median, 0.24 (IQR, 0.137–0.358) vs. 0.096 (IQR, 0.063–0.129); *p* < 0.001]. The most common location of FAs was the superior cerebellar artery (32%), while the least common location was the basilar artery branches (8%). The distribution of aneurysm locations was similar between these two groups. The aspect ratio did not show a significant difference [median, 1.16 (IQR, 1.03–1.85) vs. 1.17 (IQR, 1.00–1.48); *p* = 0.506].

### Multivariable analysis

In univariable analysis, five variables, namely AVM size, Spetzler–Martin grade, aneurysm size, SR, and RSR, that demonstrated potential significance were considered for inclusion in the multivariable logistic regression model. To avoid substantial collinearity between these variables, collinearity analysis was conducted (Supplementary Table 1). Variables such as aneurysm size (VIF = 32.76) and RSR (VIF = 33.23) were removed from subsequent multivariable analysis due to concerns about multicollinearity. AVM nidus size [OR, 0.92 (95% CI, 0.84–0.99); *p* = 0.039] and SR [OR, 1.66 (95% CI, 1.07–2.59); *p* = 0.024] remain significant risk factors for FA rupture (Table 3).

**Table 3:** Multivariable logistic regression.

Parameters	*b*-value	Wald	*p*-value	OR (95% CI)
AVM size	−0.087	4.256	0.039^a^	0.92 (0.84–0.99)
SR	0.509	5.12	0.024^a^	1.66 (1.07–2.59)
Spetzler–Martin grading	−0.241	0.198	0.656	0.79 (0.27–2.27)

OR, odds ratio; 95% CI, 95% confidence interval, SR, size ratio.

a Significant variables (*p* < 0.05).

### Treatment and outcome

Out of 37 patients, 27 (64.9%) received treatment for concurrent lesions during the initial therapy, while the remaining were treated for the ruptured lesion first. The average follow-up duration was 27.97 ± 17.03 months. Both treatment strategies yielded similar clinical outcomes. One patient had a hemorrhage, and another patient had an acute ischemic stroke immediately after AVM embolization, both which were highly related to the surgery. No novel hemorrhagic or infarct foci were found during follow-up (Table 4).

**Table 4:** Treatment and outcome.

Parameters	Total (*n* = 37)	Ruptured FAs (*n* = 22)	Unruptured FAs (*n* = 15)	*p*-value
Target of initial therapy (%)				0.006^a^
FAs	8 (21.6)	7 (31.8)	1 (6.7)	
AVMs	5 (13.5)	0 (0)	5 (33.3)	
Combined	24 (64.9)	15 (68.2)	9 (60.0)	
Acute ischemia postsurgery (%)	1 (2.7)	0 (0)	1 (6.7)	0.405
Acute hemorrhage postsurgery (%)	1 (2.7)	0 (0)	1 (6.7)	0.405
Pretreatment mRS, mean (SD)	1.57 (1.09)	1.55 (1.10)	1.60 (0.97)	0.876
Follow-up duration, month, mean (SD)	27.97 (17.03)	29.95 (19.06)	25.07 (16.34)	0.423
Follow-up mRS, mean (SD)	0.59 (0.58)	0.55 (0.64)	0.67 (0.52)	0.641
Novel ischemia during follow-up (%)	0 (0)	0 (0)	0 (0)	
Novel hemorrhage during follow-up (%)	0 (0)	0 (0)	0 (0)	

a Significant variables (*p* < 0.05).

## Discussion

A comparative analysis of demographic and clinical information and angioarchitectural and morphological characteristics in pAVMs with associated ruptured or unruptured FAs was conducted in this study. The overall incidence of FAs in pAVMs in this study was 32.7% (37/113), which is modestly higher than the previously reported range of 11.2–25% (4, 15). No significant difference was observed in the mean age at presentation between the groups. Sex, hypertension, and diabetes were not associated with FA rupture. AVM nidus size [OR, 0.92 (95% CI, 0.84–0.99); *p* = 0.039] and SR [OR, 1.66 (95% CI, 1.07–2.59); *p* = 0.024] are independent risk factors for FA rupture in multivariable logistic regression. Although the Spetzler–Martin grade of AVMs, aneurysm size, and RSR did not retain statistical significance in the multivariable regression analysis, numerically significant differences were observed between the groups. Further studies are warranted to elucidate their pathophysiological roles in FA rupture.

FAs are more common in pAVMs than those located in the anterior circulation (11). Contrary to the associated venous aneurysm, which is considered a protective factor against AVM rupture, concurrence of FAs increases the risk of hemorrhage, and FA rupture is the main reason for hemorrhage, approximately accounting for 49.2% of cases (2, 16). AVMs located in the cerebellar vermis and hemispheres are more likely to accompany FAs (62.2%). Previous studies reported that FAs in smaller AVMs tend to rupture; this conclusion aligns with our study (11, 17). Smaller AVMs appear to have elevated blood pressure in the feeding artery, which may be the reason for FA rupture (16). Although Zhang et al. (16) demonstrated a significant correlation between smaller AVMs and hemorrhagic incidents, the ruptured AVM, which is contained in the unruptured FA group, is larger in our study. This discrepancy may be attributed to two factors: first, the previous studies included AVMs supplied by both anterior and posterior circulations; second, there was no significant difference in the distribution of intranidal or flow-related aneurysms between the ruptured and unruptured AVM groups, which led to their exclusion from model construction. Soldozy et al. (18) demonstrated that ruptured aneurysms tended to have higher wall shear stress (WSS) and wall shear stress gradient associated with lower oscillatory shear index, which represented faster, impinging flow. In coronary artery diseases, several parameters, such as mean time average peak velocity and mean diastolic/systolic velocity ratio, are used to evaluate pre- and post-procedural variations using Doppler guidewires (19). In contrast, the field of cerebrovascular disease predominantly relies on transcranial Doppler ultrasound, with very few reports on the use of intravascular Doppler ultrasound; therefore, further investigation is needed to explore the links between intravascular pressure changes, blood flow velocity parameters, and hemodynamic parameters in the cerebrovascular system.

Hypertension, smoking, alcohol consumption, and being female have been reported as risk factors for intracranial aneurysm rupture (8, 20). However, there were no significant differences in our study. Several predictors of intracranial aneurysm rupture, including AR, SR, bifurcation angle, and WSS, have been demonstrated in previous studies (10, 13, 21). We found that ruptured FAs had a larger SR and aneurysm size compared to unruptured FAs [median, 2.72 (IQR, 1.95–4.12) vs. 2.23 (1.10–2.87); median size, 4.55 mm (IQR, 3.20–7.55 mm) vs. 3.00 mm (IQR, 2.40–5.00 mm), respectively]. However, the AR did not differ significantly [median, 1.16 (IQR, 1.03–1.85) vs. 1.17 (IQR, 1.00–1.48)]. Aneurysm wall inflammation is positively related to morphological characteristics. Specifically, intracranial aneurysms with larger size and size ratio exhibit increased expression of CD68 and NFKB1, which reflect acute inflammation and the activation of inflammatory pathways, respectively. Elevated levels of inflammation may lead to aneurysm wall remodeling, which is associated with rupture (22).

It is generally agreed that the ruptured lesion should be prioritized for treatment in cases involving hemorrhagic complications (2). However, the timing for treating a concurrent lesion is still debated. Redekop et al. (1) and Andereggen et al. (23) reported spontaneous regression of FAs following the treatment of associated AVMs. Świątnicki et al. (24), meanwhile, reported the growth of FAs after AVM occlusion. In our study, the majority of patients were treated for concurrent lesions at initial therapy, while others were treated for the ruptured lesion first. Only two patients experienced surgery-related hemorrhagic or ischemic events. The outcomes of both strategies during follow-up were similar. Considering the risk factors related to FA rupture, FAs in smaller AVMs or with a larger SR should be treated early.

There exist several limitations in this retrospective study. One of the limitations of this study is that only 37 patients were ultimately included in the analysis, which is insufficient to accurately elucidate the relationship between disease characteristics and aneurysms. Subsequent studies should include cases from multiple centers to validate the findings presented here and to enhance the generalizability of the results. In addition, the retrospective nature of this article makes it challenging to draw unequivocal conclusions regarding the rupture risk. Previous reports demonstrated that hemodynamic characteristics are also associated with aneurysm status. In this study, we focus on demographic, angioarchitectural, and morphological characteristics, and further research is needed to clarify the relationship between FA rupture and hemodynamic characteristics.

## Conclusion

Risk factors for FAs associated with pAVMs remain largely unknown. In this study, we focus on the demographic, angioarchitectural, and morphological characteristics of pAVMs and FAs. Our findings indicate that a smaller AVM size in maximum diameter and a larger size ratio are independent risk factors for FA rupture.

## Data Availability

The raw data supporting the conclusions of this article will be made available by the authors, without undue reservation.
