# Supplementary material for: Risk factors for flow-related aneurysm rupture associated with posterior circulation arteriovenous malformation: a multicenter retrospective study
Source: Front Neurol. 2025 Nov 24;16:1685261. doi: 10.3389/fneur.2025.1685261 (PMC12682677; doi:10.3389/fneur.2025.1685261)
Supplement: Supplementary file 1 [file Table_1.docx]

**Supplementary table 1. Multicollinearity analysis**

| **Parameters** | **VIF** | **Tolerance** | **Decision** |
| --- | --- | --- | --- |
| AVM size | 4.912 | 0.204 | retained |
| Aneurysm size | 32.76 | 0.031 | excluded |
| SR | 1.552 | 0.644 | retained |
| RSR | 33.23 | 0.03 | excluded |
| Spetzler–Martin grading | 2.32 | 0.43 | retained |
| **VIF: variance inflation factors** | | | |

**Supplementary table 2. Hemorrhagic presentation**

|  | **Total** | **None** | **SAH** | **ICH** | **IVH** | **Mixed** |
| --- | --- | --- | --- | --- | --- | --- |
| Ruptured FA | 22 | 0 | 22 | 6 | 5 | 11 |
| Unruptured FA | 15 | 2 | 4 | 13 | 1 | 5 |
| SAH:subarachnoid hemorrhage; ICH: intracerebral hemorrhage; IVH: intraventricular hemorrhage;  Mixed: presented with two or more types of hemorrhage | | | | | | |
